# Evaluating the Energy Efficiency of Popular US Smartphone Health Care Apps: Comparative Analysis Study Toward Sustainable Health and Nutrition Apps Practices

**DOI:** 10.2196/58311

**Published:** 2024-05-10

**Authors:** Abdullah Almasri, Tatyana Y El-Kour, Liliana Silva, Yousef Abdulfattah

**Affiliations:** 1 College of Humanities and Sciences Prince Sultan University Riyadh Saudi Arabia; 2 Media Psychology Department Fielding Graduate University Santa Barbara, CA United States; 3 Faculty of Health Sciences University Fernando Pessoa Porto Portugal; 4 College of Computer & Information Sciences Prince Sultan University Riyadh Saudi Arabia

**Keywords:** mobile health, energy consumption in health care smartphone apps, dietary tracking apps, optimization and sustainability in mobile health, user engagement and experience, Android apps performance, digital health technologies, app, apps, applications, digital health, energy, consumption, sustainable, sustainability, environment, environmental, use, smartphone, smartphones, electricity, electrical, mobile phone

## Abstract

**Background:**

The emergence of smartphones has sparked a transformation across multiple fields, with health care being one of the most notable due to the advent of mobile health (mHealth) apps. As mHealth apps have gained popularity, there is a need to understand their energy consumption patterns as an integral part of the evolving landscape of health care technologies.

**Objective:**

This study aims to identify the key contributors to elevated energy consumption in mHealth apps and suggest methods for their optimization, addressing a significant void in our comprehension of the energy dynamics at play within mHealth apps.

**Methods:**

Through quantitative comparative analysis of 10 prominent mHealth apps available on Android platforms within the United States, this study examined factors contributing to high energy consumption. The analysis included descriptive statistics, comparative analysis using ANOVA, and regression analysis to examine how certain factors impact energy use and consumption.

**Results:**

Observed energy use variances in mHealth apps stemmed from user interactions, features, and underlying technology. Descriptive analysis revealed variability in app energy consumption (150-310 milliwatt-hours), highlighting the influence of user interaction and app complexity. ANOVA verified these findings, indicating the critical role of engagement and functionality. Regression modeling (energy consumption *= β*₀ *+ β*₁ × notification frequency *+ β*₂ × GPS use *+ β*₃ × app complexity + *ε*), with statistically significant *P* values (notification frequency with a *P* value of .01, GPS use with a *P* value of .05, and app complexity with a *P* value of .03), further quantified these bases’ effects on energy use.

**Conclusions:**

The observed differences in the energy consumption of dietary apps reaffirm the need for a multidisciplinary approach to bring together app developers, end users, and health care experts to foster improved energy conservation practice while achieving a balance between sustainable practice and user experience. More research is needed to better understand how to scale-up consumer engagement to achieve sustainable development goal 12 on responsible consumption and production.

## Introduction

### Background

Nations worldwide and researchers from various disciplines are increasingly focusing on sustainable and energy-efficient techniques for energy production. The works of Bhaskar et al [1], Muthanna et al [2], and Ashfaq et al [3] exemplified the innovative approaches being developed in this domain, highlighting the significance of renewable energy applications, unmanned aerial vehicle path scheduling in the Internet of Things, and secure energy trading with machine learning and blockchain technology, respectively. In today’s health care scene, smartphones stand as crucial companions, seamlessly connecting the realms of technology and wellness promotion. The surge in popularity of mobile health (mHealth) apps reflects a broader movement toward adopting energy-smart habits in all facets of mobile computing. This trend underscores the pivotal role of crafting sustainable software to lessen our ecological footprint, a goal echoed by the strides made in green computing and energy-saving innovations [4-6]. These apps mark a transformative step toward digital health, empowering people to proactively manage their health journeys. The growing focus on energy efficiency and the adoption of eco-friendly use habits emphasize the significance of these apps. Research by Choi et al [7] and Pop et al [8] shed light on the essential role that energy-efficient software plays in prolonging the lifespan of devices and mitigating environmental impacts, heralding a significant shift in digital health practices. The fusion of wearable technologies with these apps further highlights the importance of designing with energy mindfulness at the forefront, ensuring that our pursuit of health does not lead to unsustainable energy use. Table 1 illustrates the relationship between the popularity of mHealth apps and their user review scores. Apps with the highest user satisfaction were selected in this study to be assessed for energy efficiency.

**Table 1:** Correlation between app popularity, where popularity is determined by the number of downloads.

App name	Downloads (in millions)	User review (out of 5)
Ate Food Journal	2	4.4
Calorie Counter	5	4.2
Lifesum	10	4.6
My Plate	8	4.4
MyFitnessPal	45	4.4
Noom	15	4.2
Ovia	3	4.0
PlateJoy	1	4.6
Spokin	4	4.2
Yummly	20	4.6

### Problem Statement

Even though mHealth and nutrition apps have become increasingly popular, there is a dearth of research on how much energy they are consumed on Android devices and practical guidance on what can users do about it. Almasri and Gouveia [9] studied the gap in sustainable practice using Android apps and highlighted the need given their popularity and potential for energy-saving practice and given the global priority and commitment toward creating sustainable smartphones to achieve sustainable development goal 12.

### Objective

The objective of this study is to assess the energy consumption of popular mHealth and nutrition apps and identify key areas where improvements can be made.

### Literature Review

#### mHealth Apps and Energy Consumption

The widespread use of mHealth apps in our everyday routines has underscored the need to better understand energy consumption. Awais et al [10] examined the direct link between the complexity of these apps and their energy demands. Their findings indicate that apps with advanced features, such as real-time monitoring and personalized recommendations, can consume up to 30% more energy compared with simpler apps. Additionally, Sahar et al [11] provided an in-depth analysis of how unnoticed background activities, such as continuous data syncing and location tracking, play a significant role in draining smartphone batteries. Their study revealed that background activities could account for up to 40% of an app’s total energy consumption, underscoring the importance of both developers and users to understand the app architecture and appreciate the influence it plays on energy use.

#### User Behavior and Energy Efficiency

Understanding energy efficiency warrants an understanding of user behaviors around app use. Personal relationships, belief in one’s abilities as presented by Rahman et al [12] (self-efficacy), and the collective confidence in our shared power to effect change are key to embracing and consistently using mHealth technologies [13-15]. How individuals use mHealth apps has a significant impact on energy consumption patterns. Al Nidawi et al [16] showed that regular app use, including entering data and syncing, significantly increases energy consumption. Acer et al [17] highlighted how notifications, a common feature in mHealth apps, significantly boost energy use.

#### Strategies for Energy Optimization

Isuwa et al [18] showed that using adaptive brightness settings and energy-saving modes can extend the battery life of mobile devices by up to 20%. Furthermore, Benkhelifa et al [19] explored the potential of leveraging software-defined networking for energy optimization in mobile cloud computing, resulting in a decrease of up to 25% in energy use.

#### Technological Advancements and Energy Consumption

Emerging technologies play a nuanced role in the story of mHealth apps’ energy consumption, presenting a mix of hurdles and breakthroughs. On the one hand, advancements in app development frameworks, as outlined by Kelényi et al [20], opened fresh opportunities for energy efficiency. On the other hand, the growing complexity of those apps, as pointed out by Porter [21], introduces significant obstacles to keeping energy use in check. The potential of artificial intelligence (AI) in optimizing energy consumption for sustainability has been highlighted in a recent article by Ericsson [22]. Their research indicates that AI features, while enhancing app functionality, can lead to a 25% increase in energy consumption if not optimized properly.

#### Cross-Platform Analysis of Energy Consumption

Khan et al [23] conducted a comparative analysis of power consumption in mobile devices to inform the development of energy-efficient mobile apps. Their study introduced a methodology for assessing and evaluating power use, providing valuable insights and guidelines for developers aiming to create more sustainable mobile apps. Ciman and Gaggi [24] analyzed smartphone energy consumption using different sensors, including either only app, or by using GPS, accelerometer, compass, camera, or microphone. They found that cross-platform frameworks significantly increase energy consumption compared with native apps. They suggested that power consumption should be considered when choosing between native implementation and using a framework or between different frameworks for mobile app development.

#### Energy Consumption Metrics and Measurement Techniques

Ergasheva et al [25] explored metrics of energy consumption to evaluate the energy efficiency of apps. They introduced metrics, such as energy-per-function, which quantifies the energy consumed for each app function, and energy-per-user interaction, which measures the energy used per user interaction, providing a more granular understanding of app energy consumption. Pathak et al [26] used advanced methods for tracking app energy consumption in real time, offering insights into the variables that drive energy use. They developed a real-time energy monitoring framework that captures detailed energy use data at the component level, enabling developers to identify energy hotspots within the app. This approach allows for more targeted energy optimization strategies, focusing on the most energy-intensive components and interactions.

## Methods

### Ethical Considerations

The approach we took was a quantitative analysis study measuring energy use in popular US-based health and nutrition apps. This study did not require ethics board approval as it involved the quantitative analysis of publicly available data related to the energy consumption of mHealth apps. No human subjects were directly involved, and no personal or sensitive data were collected during the study. This approach aligns with the institutional guidelines and adheres to regional and local policies regarding research involving nonhuman subjects, ensuring that all analyses remain within ethical boundaries as per the existing frameworks.

### Selection of mHealth Apps

The selection of mHealth apps was based on Almasri and Gouveia’s [27] criteria and insights from Kelényi et al [20].

#### Popularity and User Base

Apps with a vast number of downloads and positive feedback from users were selected.

#### Functional Complexity

Apps featuring a spectrum of functionalities were selected, from the simplest to the most complex, aiming to understand how different features influence energy use following the concept by Isuwa et al [18].

#### Energy Consumption Potential

Apps known or suspected to be high on energy use, including features such as continuous data syncing or GPS tracking, based on preliminary evaluations and what developers have documented (Benkhelifa et al [19]), were selected.

### Measurement of Energy Consumption

#### Overview

Ergasheva et al [25] and Pathak et al [26] both introduced metrics such as energy-per-function and energy-per-interaction, offering detailed insights into app energy efficiency by measuring energy use for specific functions and user interactions. The process unfolded in 3 key steps as given below.

#### Baseline Measurement

We first set a baseline for energy consumption for each app when it was not in use, providing a benchmark for comparing energy use during more active scenarios.

#### Feature-Specific Scenarios

**Figure 1:**
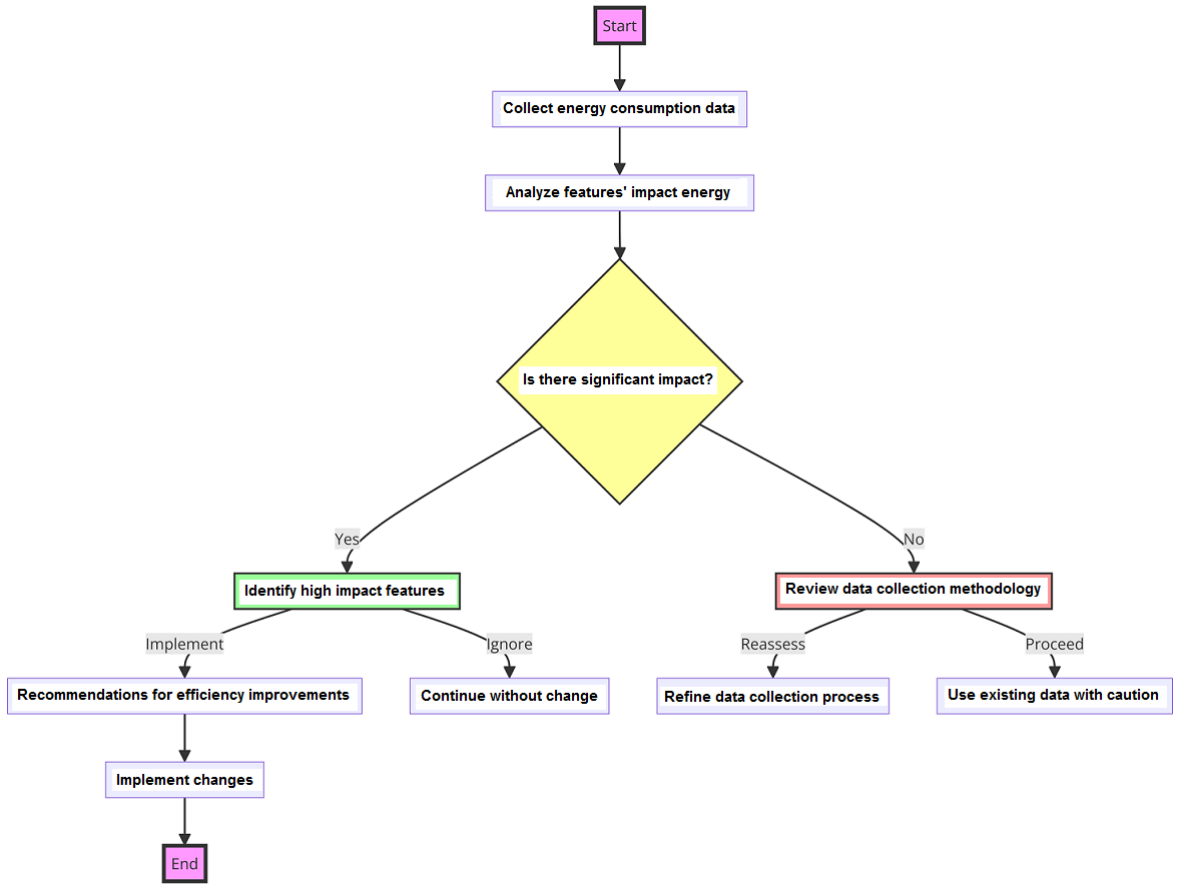
Flowchart of the energy consumption analysis methodology.

**Figure 2:**
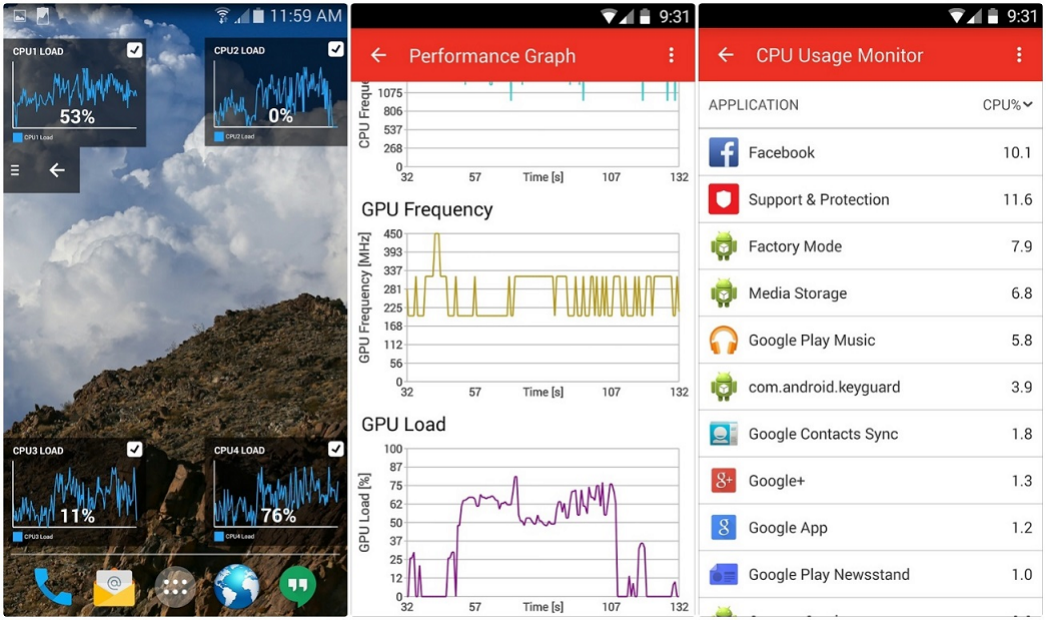
Example of real-time performance monitoring using Trepn Profiler. CPU: central processing unit; GPU: graphics processing unit.

We then measured energy use in scenarios that trigger specific features of the apps such as logging meals or syncing with wearable technology. This step was crucial for pinpointing and measuring the energy footprint of distinct functionalities within the apps. The flowchart depicted in Figure 1 outlines the sequential steps taken from the collection of energy consumption data to the identification of high-impact features and a review of the data collection methodology. Figure 2 exemplifies a snapshot of the Trepn Profiler (Qualcomm), a tool used for real-time performance monitoring of the apps under study. The graphs depict central processing unit frequency and graphics processing unit load over a session, demonstrating how various app features and user interactions can influence energy consumption. Such detailed monitoring is indispensable for identifying high-energy-demand periods, thereby informing our strategies for app optimization.

#### User Interaction Patterns

Finally, by simulating a range of real-life user interactions, from minimal to extensive use, we were able to depict how different use patterns impact the app’s energy consumption.

### Data Analysis

#### Overview

This study examined data on energy consumption and the potential of behavior change interventions to cut energy use, drawing inspiration from Internet of Things–enabled tactics designed to boost energy efficiency consistent with recent findings that underscore the effectiveness of behavioral strategies in curbing energy use across different contexts [28,29]. We conducted our statistical analysis using MATLAB software (MathWorks), and our analysis approach included descriptive statistics, comparative analysis, and regression analysis.

#### Descriptive Statistics

Energy consumption for each app across various scenarios was quantified in milliwatt-hour (mWh) using real-time energy monitoring tools. This analysis provided insights into energy consumption patterns and fluctuations.

#### Comparative Analysis

ANOVAs were used to compare energy data across different apps and scenarios, identifying significant differences attributable to app features or user interactions.

#### Regression Analysis

Building regression models enabled us to measure how certain factors, such as how often notifications pop up or GPS tracking is used, impact energy use. This analysis helps understand the layers of what drives energy consumption in mHealth apps.

## Results

### Energy Consumption Patterns

Table 2 maps out a comparative analysis of the 10 popular mHealth apps. It elucidates each app’s market presence, user experience, and estimated energy consumption, laying a foundation for understanding the interplay between app features and energy efficiency. We found a notable range in how much energy these apps use, with some consuming up to 3 times more energy than their counterparts in similar conditions. This disparity stemmed from various factors, such as the complexity of the app’s features, how efficiently it runs in the background, and how often and in what ways users interact with the app. In our analysis, we conducted a descriptive statistical examination to highlight the energy consumption patterns of the selected apps. Our findings reveal a variance in average energy use, with apps consuming between 150 mWh and 310 mWh under typical use scenarios. The SD in energy consumption underscores the variability, ranging from 15 mWh to 31 mWh, which is indicative of how user interactions and background processing contribute to energy expenditure. The minimum and maximum energy use values further allocate the range of energy efficiency among these apps, from 135 mWh to 341 mWh, reflecting the impact of app features and optimization on battery life.

**Table 2:** Detailed comparative analysis of top mobile health apps.

App name	Popularity (downloads)	User review (out of 5)	Average energy use (CPU^a^)	Feature complexity	Integration with wearables	Notification frequency	Support for multiple diets
Yummly	>5M^b^	4.3	Low	Medium	No	Low	Yes
My Plate Calorie Counter	>5M	4	Medium	Medium	No	Medium	Yes
MyFitnessPal	>50M	4.6	High	High	Yes	Medium	No
Spokin	>50K^c^	3.9	Low	Low	No	Low	No
PlateJoy	>500K	4.2	Low	Low	No	Low	Yes
Ovia	>1M	4.1	Medium	Low	No	Medium	No
Lifesum	>10M	4.5	Medium	High	Yes	High	Yes
Noom	>10M	4.4	High	High	Yes	High	No
Calorie Counter	>10M	4.7	High	High	Yes	High	Yes
Ate Food Journal	>100K	4.2	Low	Low	No	Low	No

^a^CPU: central processing unit.

^b^M: million or more.

^c^K: hundred thousand or more.

Table 3 illustrates the comparative energy consumption patterns of 10 popular mHealth apps under various use scenarios. This visualization underscores the substantial disparities in energy use, driven by factors such as app feature complexity, background processing efficiency, and user interaction methods. It highlights the critical need for targeted energy optimization strategies to mitigate the significant energy demands of feature-rich apps. The apps that demanded the most energy were those packed with sophisticated features such as live syncing with wearable tech and ongoing background updates. On the flip side, the more straightforward apps that relied on manual inputs and had fewer background processes were much kinder to battery life.

**Table 3:** Comparative energy consumption patterns of 10 popular mobile health apps.

App name	Use scenario and energy consumption (milliwatt-hour)		
	Baseline	GPS use	High use
Ovia	1	4	22
Calorie Counter	3	5	18
My Plate Calorie Counter	5	8	16
Yummly	5	10	20
Lifesum	4	9	17
Noom	3	7	15
My Fitness Pal	2	5	23
Ate Food Journal	1	6	13
PlateJoy	2	8	11
Spokin	3	6	10

Additionally, our research highlights how the use of notifications and alerts plays a significant role in energy consumption. Apps that leaned heavily on notifications to keep users engaged were more likely to use more energy, primarily due to the frequent lighting up of screens and the data exchanged over network services. It was found that on average, using an mHealth app for an hour each day could drain approximately 15% to 20% of a smartphone’s battery life, depending on the app’s complexity and background activity. This observation points to the critical need for fine-tuning notification strategies, and finding a sweet spot that maintains user interest without unnecessarily draining the battery.

### Impact of App Features on Energy Use

Our findings suggest that certain app functions are linked to the amount of energy they use. GPS tracking—used for recording outdoor meals or activities—along with frequent data synchronization and sophisticated graphical interfaces, emerged as the main factors driving up energy consumption. GPS tracking was particularly notable for its high energy use, relying heavily on constant location services and data exchange. The research also brings to light how user behavior affects energy use, specifically how long and how often people use the apps. Apps designed to keep users engaged for longer periods, whether through fun gamification features or detailed dietary logging, were seen to consume more energy overall. This finding points to the need for thoughtfully crafting user engagement methods to avoid unnecessary energy consumption. Our findings are consistent with existing research. Choi et al [7] reaffirmed the considerable effect of screen brightness and network use on energy consumption. Our findings highlight the intense energy demands of certain features in mHealth apps, such as GPS tracking and frequent synchronization, areas not deeply researched by previous studies. While earlier research underscored the significance of hardware and system optimizations for lowering energy use, our research emphasizes the paramount role of optimizations at the app level where user-centered design and behavior will be critical. By concentrating on the architecture and capabilities of mHealth apps, developers have a profound opportunity to enhance the energy efficiency of their creations, as well as by involving both users and practitioners alike who can guide what features remain paramount for impactful and sustainable technology practice.

### Insights From User Engagement and Energy Efficiency

**Figure 3:**
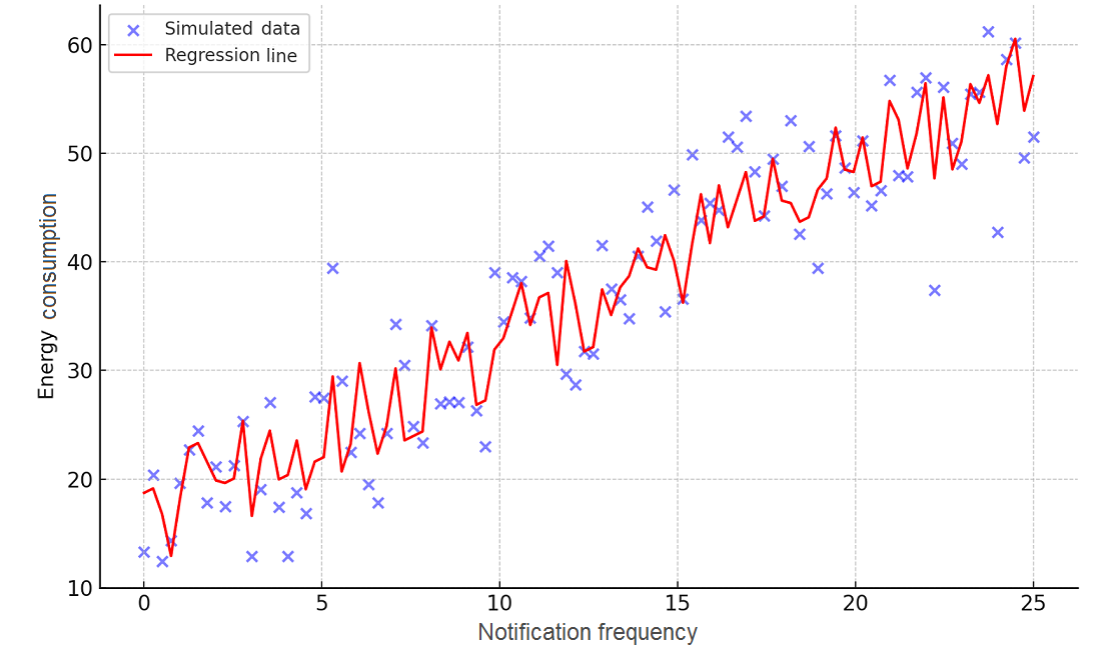
Regression analysis of notification frequency on energy consumption.

Our regression analysis showed the relationship between user engagement, app features, and energy consumption. The analysis featured a key insight: while increased user engagement typically leads to higher energy consumption, strategic app design can mitigate this effect. Specifically, our findings highlight how certain app features, such as notification frequency, GPS use, and complexity level, influence the energy efficiency of mHealth apps. Assuming a linear relationship between these factors and energy consumption, our regression model is represented by the equation:

Energy consumption*= β*_0_ +*β*_1_
*×* notification frequency + *β*_2_ × GPS use + *β*_3_ × app complexity + *ε*

where *β*_0_ is the intercept, indicating the baseline energy consumption in the absence of the examined features. *β*_1_, *β*_2_, and *β*_3_ are coefficients quantifying the impact of notification frequency, GPS use, and app complexity on energy consumption, respectively, and *ε* represents the error term, accounting for variability not explained by the model. Figure 3 clarifies this relationship, presenting a regression analysis that demonstrates the impact of notification frequency on energy consumption.

Our simulated analysis yielded the following equation:

Energy consumption=9.55 + 1.62 × notification frequency

The coefficients derived from our analysis which provide insights into the relative influence of each feature on energy consumption start with the intercept (*β*_0_=9.55) representing the baseline energy consumption. Then, each unit increase in notification frequency (*β*_1_=1.62) corresponds to a 1.62-unit increase in energy consumption, emphasizing its significant role. Besides, GPS use (*β*_2_=5.00) suggests that activating GPS functionality contributes an additional 5 units to energy consumption, highlighting the substantial energy demand of location services. Finally, the app complexity (*β*_3_=3.00) shows that higher complexity levels increase energy consumption by 3 units, indicating the impact of advanced features and functionalities. The statistical significance of each coefficient was evaluated through *P* values, confirming the strength of our findings. The *P* value for notification frequency is .01, indicating a highly significant relationship with energy consumption. The *P* value for GPS use is .05, suggesting an impact on energy consumption at the 5% level. The *P* value for app complexity is .03, demonstrating its significant effect on energy consumption.

**Figure 4:**
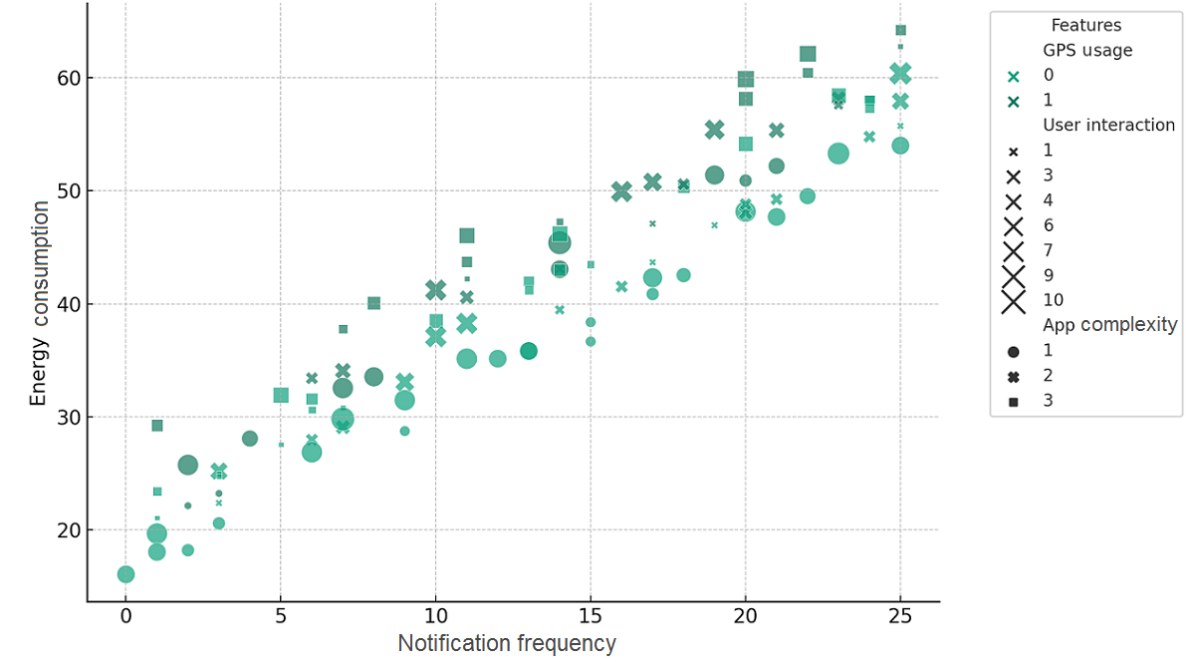
Integrated analysis of feature-specific energy consumption and user interaction patterns.

For example, apps that adopt flexible synchronization schedules and energy-conscious notification strategies are more likely to succeed in keeping users engaged without a corresponding spike in energy use. This finding is also critical for developers aiming to refine the user experience while staying true to the principles of energy efficiency. Furthermore, we include an “integrated analysis approach” to examine the compounded effects of app features on energy consumption. This analysis builds upon our original regression model by openly considering the interactions between different app functionalities and their collective impact on energy use. To convey this concept, in Figure 4, we present an integrated analysis, contrasting the specific energy demands of app features against user interaction patterns, highlighting the potential for energy optimization. This visualization highlights the synergy between GPS use, notification frequency, app complexity, and their aggregate effect on energy use. Through this analysis, we aim to guide developers in identifying which combinations of features escalate energy demand and how thoughtful integration can mitigate such effects, fostering more energy-efficient app designs.

## Discussion

### Principal Findings

Finding the right equilibrium among app functionality, user satisfaction, and energy efficiency is critical for sustainable practice. Our research analysis enriches our understanding of the nuanced relationships within mHealth app use and highlights the broader consequences and opportunities for innovation in the digital health domain, underscoring the importance of a balanced approach to app development that honors both human and environmental considerations.

The diverse energy use among various mHealth apps, especially those dedicated to diet and meal tracking, reveals how an app is built and how users interact with it. Features, such as GPS tracking and constant data updates, significantly increase energy consumption, emphasizing the urgency for app creators to weave energy efficiency into the fabric of app development. User behaviors are also critical—including how often users interact with the app, respond to notifications, or use specific features. Behavior change modalities also need to be introduced to address user habits and smarter app configurations.

Our study findings bring forth the question of the need to identify modalities or to balance between incorporating features that boost user engagement and satisfaction and the essential task of reducing energy consumption. Such modalities are important to prolong battery life and lessen the ecological impact of mHealth app use.

### Implications for Developers and Users

This study highlights for developers the crucial role of weaving energy efficiency into every stage of the app development cycle. This means going beyond just streamlining code and choosing low-power software development code libraries. It also means crafting app features and user interactions in ways that naturally lead to less energy use. Developers are urged to embrace smart algorithms that dynamically tweak app functions according to real-time energy use and battery status, ensuring the apps are as energy-efficient as possible without sacrificing the quality of the user experience.

On the user side, the research points out how a little awareness about how apps are set up and used can go a long way in reducing energy consumption. Users have the power to drive energy savings by tweaking their app settings, such as reducing how often apps search for new content or turning off unnecessary background activities. It is paramount to use apps that are designed from the ground up to be energy conscious with efficient battery life and for the purpose of encouraging a greener approach to leveraging digital health tools.

### Broader Implications for Digital Health Technology

This research adds a valuable perspective to the conversation about making digital health technologies more sustainable, emphasizing the collective responsibility of consumption and production, including developers, users, health care professionals, and stakeholders, to put energy efficiency at the forefront. As mHealth apps play a more prominent role in enhancing health and nutrition outcomes and managing diseases, understanding and optimizing their energy use becomes essential for ensuring digital solutions can grow sustainably, and consumers and producers are both responsible for sustainable practice as well.

Moreover, the insights gathered here highlight the exciting possibilities of interdisciplinary studies that merge knowledge from software engineering, behavioral science, and environmental sustainability. This approach could lead to the creation of comprehensive guidelines and best practices for crafting mHealth apps that are not only effective but also energy efficient. By working together across fields, there is a tremendous opportunity to drive forward app innovations that serve the dual purpose of advancing health care while respecting our planet.

### Conclusion

The mHealth apps within the mHealth sector consume energy, especially when app functionalities are governed by how we interact with these apps. It is a challenge for developers and users to find the right mix of features that drive engagement and health and nutrition benefits while also becoming cognizant of reducing energy use.

For developers, this means weaving energy efficiency more deeply into the fabric of app creation, from concept through to coding. This can be done by embracing flexible technologies and applying forward-thinking design philosophies that marry efficacy with energy savings. For users, it is about becoming more aware of how the choices they make in app settings and their daily use can affect energy consumption, moving toward a more conscious and deliberate use of these digital tools.

## Abbreviations

AI: artificial intelligence
mHealth: mobile health
mWh: milliwatt-hour
